# Ocean connectivity and habitat characteristics predict population genetic structure of seagrass in an extreme tropical setting

**DOI:** 10.1002/ece3.10257

**Published:** 2023-07-03

**Authors:** Udhi E. Hernawan, Kor‐jent van Dijk, Gary A. Kendrick, Ming Feng, Oliver Berry, Christopher Kavazos, Kathryn McMahon

**Affiliations:** ^1^ School of Science and Centre for Marine Ecosystems Research Edith Cowan University Joondalup Western Australia Australia; ^2^ Research Centre for Oceanography (PRO), National Research and Innovation Agency (BRIN) Jakarta Indonesia; ^3^ School of Biological Sciences The University of Adelaide Adelaide South Australia Australia; ^4^ School of Biological Sciences and The Ocean Institute The University of Western Australia Crawley Western Australia Australia; ^5^ Western Australian Marine Science Institution Perth Western Australia Australia; ^6^ CSIRO Environment, Indian Ocean Marine Research Centre Crawley Western Australia Australia

**Keywords:** extreme tidal range, gene flow, local adaptation, oceanographic connectivity, seascape genetics, *Thalassia hemprichii*

## Abstract

Understanding patterns of gene flow and processes driving genetic differentiation is important for a broad range of conservation practices. In marine organisms, genetic differentiation among populations is influenced by a range of spatial, oceanographic, and environmental factors that are attributed to the seascape. The relative influences of these factors may vary in different locations and can be measured using seascape genetic approaches. Here, we applied a seascape genetic approach to populations of the seagrass, *Thalassia hemprichii*, at a fine spatial scale (~80 km) in the Kimberley coast, western Australia, a complex seascape with strong, multidirectional currents greatly influenced by extreme tidal ranges (up to 11 m, the world's largest tropical tides). We incorporated genetic data from a panel of 16 microsatellite markers, overwater distance, oceanographic data derived from predicted passive dispersal on a 2 km‐resolution hydrodynamic model, and habitat characteristics from each meadow sampled. We detected significant spatial genetic structure and asymmetric gene flow, in which meadows 12–14 km apart were less connected than ones 30–50 km apart. This pattern was explained by oceanographic connectivity and differences in habitat characteristics, suggesting a combined scenario of dispersal limitation and facilitation by ocean current with local adaptation. Our findings add to the growing evidence for the key role of seascape attributes in driving spatial patterns of gene flow. Despite the potential for long‐distance dispersal, there was significant genetic structuring over small spatial scales implicating dispersal and recruitment bottlenecks and highlighting the importance of implementing local‐scale conservation and management measures.

## Graphical abstract

Using a seascape genetic approach for the seagrass, *Thalassia hemprichii*, in a complex coastal setting with the world's largest tropical tides, we detected significant spatial genetic structure and asymmetric gene flow over small spatial scales. This pattern was explained by oceanographic connectivity and differences in habitat characteristics and highlights the importance of implementing local‐scale conservation and management measures.

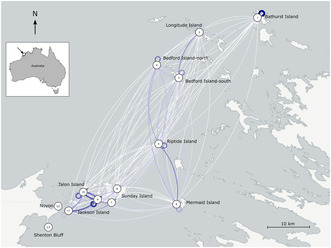

## 1 INTRODUCTION

The extent of gene flow among populations influences how genetic diversity is distributed in space (referred to as genetic structure). Understanding the genetic structure and the processes that generate them is highly informative for a broad range of conservation practices (Beger et al., 2014; Frankham et al., 2010; Magris et al., 2014; Palumbi, 2004) such as identifying conservation units (Jahnke et al., 2020; Palumbi, 2003), their spatial boundaries (e.g., Nielsen et al., 2017), and areas that are important stepping stones or the source of recruits (e.g., Coolen et al., 2020; Jahnke et al., 2018).

The geographic distance between populations, currents and ocean barriers, species dispersal capacity, and the availability of environments to colonize can all influence genetic structure. Traditionally, isolation by distance (IBD) has been the simplest model used to explain genetic differentiation, whereby gene flow decreases with distance from the original population (Wright, 1943). However, in the marine environment, genetic differentiation often cannot be explained simply as a function of geographic distance (Riginos & Liggins, 2013). Dispersal of most marine organisms can be either facilitated or limited by oceanographic features (such as water currents) regardless of the geographic distances. Therefore, patterns of genetic differentiation often follow a function of resistance to gene flow, known as “isolation by resistance” (IBR; Thomas et al., 2015) or “isolation by oceanographic distance” (IBOD; Alberto et al., 2011). Local habitat characteristics and ecological processes (e.g., competition) can also limit gene flow by preventing successful settlement and recruitment (Binks et al., 2019; Marshall et al., 2010; Orsini et al., 2013; Ranta et al., 2009). Despite high levels of migration, a population may still be genetically isolated, if settlement is restricted or post‐recruitment mortality is high. In this case, genetic differentiation follows a model of “isolation by environment”/IBE, whereby gene flow is affected by differential environmental conditions and ecological processes between populations (Wang & Bradburd, 2014).

Gene flow is dependent on the combination of these processes as described above (e.g., Sjöqvist et al., 2015) and the relative contribution of each component which varies among systems and can be measured using seascape genetics (e.g., Giles et al., 2015; Selkoe et al., 2010). A seascape genetics approach incorporates species biological traits, oceanography, habitat characteristics, and other types of data into population genetic analyses to understand processes determining gene flow in the marine environment (Riginos & Liggins, 2013).

The Kimberley coast on the Australian North West Shelf is rich in biodiversity, yet the least scientifically studied region in Australia (Wilson, 2013). It is located within one of the lowest human‐impacted regions in world (Halpern et al., 2008), and has a highly complex seascape with thousands of islands and the world's largest tropical tidal ranges of up to 11 m (Gruber et al., 2017; Wilson, 2013). The local currents are heavily influenced by tide, and override the broader scale, outer continental shelf currents (Condie & Andrewartha, 2008). Currents around the islands are multidirectional and can exceed 1 per ms, producing spectacular ocean conditions including whirlpools and standing waves (Cresswell & Badcock, 2000; Lowe et al., 2015; Wilson, 2013).

The growing interest in the industrial use of the Kimberley marine environment, expansion of tourism, impacts from climate change and the cultural significance for traditional owners (Boschetti et al., 2020; Strickland‐Munro et al., 2016) as well as a limited baseline understanding of important processes, such as genetic structuring, urge scientific investigations on this topic. In this region, the influence of ocean currents on gene flow has been examined at a large scale (100s–1000s km) where genetic structure has been identified between oceanic coral reefs and the mainland (Berry et al., 2020; Condie & Andrewartha, 2008; Underwood, 2009; Underwood et al., 2012, 2020), and southward gene flow detected, aligned with the dominant southward‐moving Holloway Current (Wilson, 2013). In the coastal zone where strong, multidirectional tidal currents flow, connectivity might be high which could homogenize the spatial distribution of genetic variation. However, recent studies on fish, gastropods, and coral in the region have demonstrated that this is not always the case. Low genetic structure and high connectivity was detected for gastropods (Berry et al., 2020) and it was an admixture zone for fish (DiBattista et al., 2017), but there was significant genetic structure at scales of 35 km for corals (Underwood et al., 2020).

Seagrasses have a variety of dispersal mechanisms that could result in genetic structure over a range of spatial scales, from <10 km up to 1000 km, but generally with distances over 100 km the genetic differentiation increases (Kendrick et al., 2012, 2017; McMahon et al., 2014). For species with buoyant fruit, gene flow is facilitated by ocean currents and windage (Jahnke et al., 2019; Ruiz‐Montoya et al., 2015; van Dijk et al., 2009), and even in species without buoyant fruits, dispersal of fragments with or without seeds can also occur and in some species these fragments are buoyant (Evans et al., 2021; McMahon et al., 2014). The receiving habitat can also influence recruitment success. Although there is limited understanding on the drivers of recruitment success, studies for other species with buoyant fruits show that settlement and establishment of seeds is more common with more complex topography, at shallower depths (<3 m) and more frequently on rocky than on sandy substrate and the presence of other species can increase establishment success (Alagna et al., 2020; Balestri et al., 2017, 2021). The type of habitat, oceanographic connectivity, historical connectivity, and location in relation to the species' range have all been shown to influence the patterns of genetic structure (e.g., Alberto et al., 2006; Hernawan et al., 2017; Olsen et al., 2013).

Here, we examined the pattern of genetic differentiation and structure over a small spatial scale ~80 km, among populations of the tropical seagrass, *Thalassia hemprichii* (Ehrenberg) Ascherson, 1871 and investigated the factors that are known to influence genetic structure (i.e., spatial distance, oceanographic connectivity, and local environmental factors). We incorporated genetic data from a panel of 16 microsatellite markers, a biophysical dispersal simulation, and environmental data into a seascape genetic approach using partial RDA analysis (Meirmans, 2015). The seagrass *T. hemprichii* is among the most widely distributed seagrass species in the Indo‐West Pacific (Short et al., 2007). It is in the middle of its range in the Kimberley (Hernawan et al., 2017), dominant in the intertidal areas (Lowe et al., 2015), and an important food source for megagrazers, like dugongs and turtles (André et al., 2005). Multiple studies have assessed the genetic structure and connectivity of *T. hemprichii* from a regional and local context (Hernawan et al., 2017; Hu et al., 2021; Jahnke et al., 2019; Nakajima et al., 2023; Nguyen et al., 2022; Wainwright et al., 2018) and identified genetic structure over regional scales of hundreds of kilometers, but also in some situations genetic structuring over small spatial scales linked to oceanographic connectivity and habitat type. The species is a useful model for characterizing gene flow in the nearshore islands of the Kimberley because water currents are the main vector for its dispersal (Kendrick et al., 2012; McMahon et al., 2014). The species has a long‐distance dispersal (LDD) potential with positively buoyant fruits traveling for 2–7 days (Lacap et al., 2002). We predict that due to the strong currents in the Kimberley, there will be low genetic structure at this spatial scale and the main driver of genetic structure for *T. hemprichii* will be oceanographic connectivity rather than distance. In conjunction with oceanographic connectivity, habitat may also influence genetic structure by influencing the success of seedling recruitment and establishment.

## 2 MATERIALS AND METHODS

### 2.1 Study site and sampling

**FIGURE 1:**
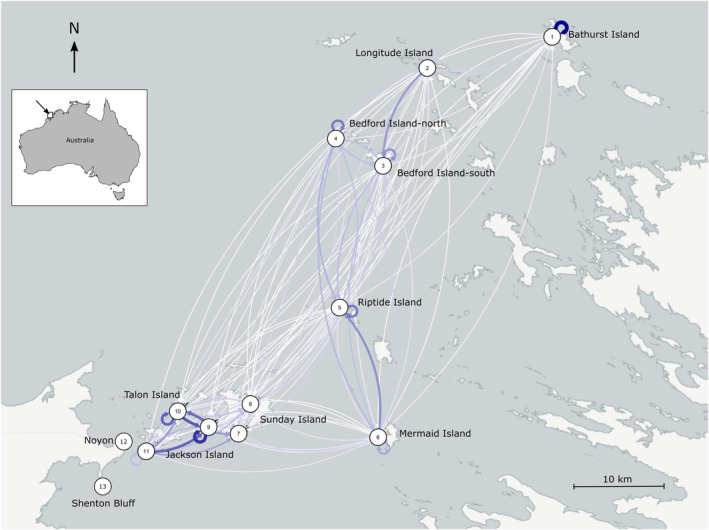
Map of the sampling sites and the pattern of oceanographic connectivity, referred to as the average number of particles released from site *i* that were tracked to be in site *j*. Connectivity among sampling sites represented by curved lines. Sampling sites (populations) were represented by numbers within circles (refer to Table 1). The thicker the lines, the higher the level of connections (number of particles) between populations.

Thirteen sites were sampled in August and October 2014 around the Buccaneer Archipelago (four sites), the Sunday Islands group including mainland sites (seven sites), and intermediate sites between the first two groups (two sites) (Table 1; Figure 1). All sampling sites were in shallow lagoonal environments where *T. hemprichii* predominantly occurs and exposed at low tide. Pairwise overwater distances between sampling sites, defined as the shortest path between two locations without crossing boundaries of any landmass (at mean sea level), ranged from 2 to 73 km. The distance calculation was performed based on high‐resolution bathymetric data from the US National Oceanic and Atmospheric Administration (NOAA) using the package *marmap* in R (Pante & Simon‐Bouhet, 2013).

**TABLE 1:** Genetic diversity of *Thalassia hemprichii* in the Kimberley obtained from 16 microsatellite loci.

Region	Site	Site ID	*N*	*G*	*R*	*nA*	*A*_R_	*P*_A_	*H*_O_	*H*_NB_	*F*_IS_	PID	PID_N_	*P*_sex_	Max. no. of samples to one MLG
Buccaneer Archipelago	Bathurst Island	1	30	14	0.48	24	1.50	0	0.232	0.168	−0.408**	<0.01	<1	2/6 (2 + 2)	6
	Longitude Island	2	48	23	0.49	30	1.83	0.06	0.291	0.216	−0.357**	<0.01	<1	0/8	12
	Bedford Island—South	3	48	37	0.77	28	1.65	0.01	0.120	0.139	0.138**	<0.05	<1	3/7 (2 + 2 + 3)	4
	Bedford Island—North	4	48	23	0.47	24	1.47	0	0.133	0.133	−0.004	<0.05	<1	5/12 (2 + 2 + 2 + 2 + 3)	7
Intermediate sites	Riptide Island	5	48	43	0.94	31	1.82	0.03	0.199	0.211	0.059*	<0.001	<1	0/4	2
	Mermaid Island	6	48	44	0.91	36	1.84	0.09	0.215	0.196	−0.097**	<0.01	<1	0/4	2
Sunday Island group, (including mainland sites)	Sunday Island—South	7	47	20	0.43	27	1.58	0.05	0.119	0.132	0.105**	<0.05	1	1/9 (3)	6
	Sunday Island—North	8	48	27	0.57	27	1.56	0.12	0.130	0.131	0.009	<0.05	1	6/10 (2 + 3 + 3 + 3 + 5 + 5)	5
	Halls Pool	9	48	32	0.66	27	1.61	0.07	0.104	0.171	0.399**	<0.01	<1	2/7 (3 + 3)	6
	Talon Island	10	48	18	0.36	31	1.84	0.12	0.208	0.180	−0.162**	<0.01	<1	0/7	16
	Jackson Island	11	48	33	0.68	31	1.73	0.08	0.135	0.141	0.047	<0.05	<1	6/11 (2 + 2 + 2 + 2 + 3 + 3)	3
	Noyon^a^	12	48	–								0.09	4		
	Shenton Bluff^b^	13	48	5	0.09							0.02	1		29
	TOTAL		557	319	0.57										

*Note*: Significance level *p < .05, **p < .01.

Abbreviations: *A*_R_, allelic richness; *F*_IS_, inbreeding coefficient; *G*, number of multilocus genotype; *H*_NB_, unbiased heterozygosity‐Nei, 1987; *H*_O_, observed heterozygosity; *N*, total number of individuals examined; *nA*, observed alleles; *P*_A_, private allele richness standardized at 20 MLGs (average alleles per loci); PID, probability of identity; PID_N_, number of samples predicted with the same MLG; *P*_sex_, number of clone mate groups where clone mates may have arisen from a sexual reproduction/all clone mate groups (*N* in clone mate group, 1 + *N* in clone mate group 2, …); *R*, clonal richness (MLG−1/*N*−1).

^a^ Markers at Noyon could not differentiate clones so this site was excluded for further analysis and not included in total.

^b^ Due to low MLG numbers at Shenton Bluff population, genetic analysis was not conducted.

At each sampling site, we recorded five variables to define environmental conditions used in the seascape genetic analysis: (i) water depth (m) relative to mean sea level (determined from Google Earth version 7.1.2.2041); (ii) dominant sediment type through visual assessment using Wentworth's categories (boulder, cobble, pebble, granule, sand, silt, and clay) (Wentworth, 1922); (iii) geomorphic habitat type (reef terrace and reef lagoon); (iv) number of other seagrass species; and (v) the presence/absence of corals (Table S1). For geomorphic habitat, reef terrace is defined as a step‐like reef flat, while reef lagoon is a shallow body of water separated from the main water body by fringing reefs.

We collected a total of 650 samples (meristematic region of seagrass leaves). At each sampling site, a total of 50 samples, separated by at least 2 m were either collected randomly in a circular area with a diameter of ~50 m and 5–10 25 m‐transects starting from the center of the circle, or if this was not possible because of the patchy nature of the meadow, then samples were collected haphazardly across the patches in a similar area. Samples were collected by wading at low tide. Due to the presence of saltwater crocodiles, it was not feasible to SCUBA dive. Clear meristematic sections of the leaf were cut into strips and then immediately stored in silica to rapidly dry and preserve the DNA. DNA was extracted from 2 to 3 pieces (5–10 mm in size) of silica dried samples using AGRF extraction service (Australian Genome Research Facility, www.agrf.org.au). Extractions were done using the Nucleospin Plant II Kit (Machery‐Nagel, Düren, Germany) with the PL2/PL3 buffer system.

### 2.2 Microsatellite amplification and genotyping

Microsatellite amplification was conducted on 16 polymorphic microsatellite loci: Thh5, Thh34, Thh15, TH66, TH37, TH43, TH43, Thh8, TH34, Thh41 TH52, TH07, Thh29, Thh1, Thh36, and Thh3 (van Dijk et al., 2014) and have been used successfully in a study from Indonesia to western Australia that incorporates some of these sites (Hernawan et al., 2017). These were amplified using fluorescently labeled primers in three separate multiplex panels with the QIAGEN Type‐it® Microsatellite PCR Kit (10‐μL reactions with ~1 ng of genomic DNA). Fragment analysis and capillary separation were run at GGF (Georgia Genomics Facility) with GGF's size standard 500 ROX. Microsatellite alleles were manually scored and checked for errors using the Microsatellite plugin v1.4 in Geneious R7 v 7.1.7 (Biomatters Ltd).

### 2.3 Genetic analysis

#### 2.3.1 Microsatellite data properties

Genotyping errors and the presence of null alleles were tested using a maximum likelihood approach implemented in ml‐nullfreq with 100,000 randomizations (Kalinowski & Taper, 2006). This has been shown to be the overall best performing method for null allele detection (Dąbrowski et al., 2015). Each sample site was assessed to test the power of the genetic marker system used to detect unique multilocus genotypes (MLGs). The probability of identity (PID), that two individuals drawn at random within a population will have the same MLG, was calculated for increasing locus combinations (Waits et al., 2001) using the program GenALEx (v6.5) (Peakall & Smouse, 2012). Then based on the number of samples collected at the site (*N*) the expected number of individuals with the same MLG was estimated. Noyon site was excluded from further analysis as the marker system was not powerful enough to detect MLGs. MLGs were identified using the package *poppr* in R (Kamvar et al., 2014). To assess if the samples assigned to the same MLG were clone mates and not generated by chance through a unique sexual reproductive event, *P*_sex_ was assessed for sets of clone mates with identical MLGs (Arnaud‐Haond et al., 2007). The maximum *N* assigned to a unique MLG was reported. For further analysis, we generated a new dataset containing only unique MLGs using the package *poppr* in R (Kamvar et al., 2014). Clonal diversity was calculated as *R* = (*G*−1)/(*N*−1) (Dorken & Eckert, 2001). Due to the low number of MLGs at Shenton Bluff, this site was excluded from further population genetic analyses.

We tested for linkage disequilibrium across multiple loci based on the standardized index of association (*r*_D_) accounting for different sample sizes using the package *poppr* (Kamvar et al., 2014). Departure from Hardy–Weinberg Equilibrium (HWE) was based on the inbreeding coefficient (*F*_IS_) calculated in Genetix 4.05 (Belkhir et al., 2004). Genetic diversity at each site was expressed as four parameters: (1) allelic richness (*A*_R_), (2) private allele richness (*P*_A_), (3) observed heterozygosity (*H*_O_), and (4) unbiased expected heterozygosity (*H*_NB_, Nei, 1987). *A*_R_ and private allele richness (*P*_A_) were calculated using HP‐Rare (Kalinowski, 2005) from a standardized set of 14 (minimum number of MLGs per site) and also 20 samples to capture more variability across samples. There was little difference in the results so the 20‐sample output was reported, but the 14‐sample output is presented in Table 1. Mean *H*_O_ and mean *H*_NB_ (Nei, 1987) were calculated using Genetix 4.05 (Belkhir et al., 2004).

#### 2.3.2 Genetic differentiation and structure

*F*_ST_ was used as a measure of genetic differentiation. Since the mutation rate can affect differentiation, the use of *F*_ST_ with highly polymorphic markers, such as microsatellites, can lead to bias in estimating genetic differentiation. The mutational effects on genetic differentiation were examined using the correlation coefficient between *G*_ST_ and *H*_S_ across loci (*r*_GH_) in the program codidi (Wang, 2015). Our *r*_GH_ was positive and not significant, inferring *F*_ST_ was not underestimated (0.2732, *p‐*value = .306, Figure S1) and that the genetic differentiation measure was not affected by mutation rate (Wang, 2015). The population‐pairwise *F*_ST_ was obtained from GenALex v6.501 (Peakall & Smouse, 2012).

Population structure was examined using a Bayesian assignment test in structure v2.3.4 (Pritchard et al., 2000). This allows us to identify the number of panmictic clusters (*K*) among the populations. We set the number of panmictic clusters (*K*) to be tested from *K* = 1 to *K* = 11, with a burn‐in of 10^5^ replications. We performed 20 iterations for each *K* value. Determining the “true” *K* was based on Evanno et al. (2005) and we used the web server Clumpak to align multiple replicate analysis of the appropriate *K*, and visualize the population structure (Kopelman et al., 2015).

#### 2.3.3 Seascape genetic analysis

##### Oceanographic connectivity

We used biophysical dispersal modeling based on Regional Ocean Modeling System (ROMS, Feng et al., 2017) with 2 km resolution to construct a site‐pairwise matrix of oceanographic connectivity. The model was nested within the Ocean Forecasting Australia Model 3 (OFAM3) simulation (Feng et al., 2016) and forced by three‐hourly meteorological measures derived from Kobayashi et al. (2015). The model simulation occurred over the 2009–2012 time period. Hourly sea surface current velocities (0–5 m) were extracted from the model output and used for particle tracking modeling. One hundred particles were seeded at each seagrass sampling sites during austral spring–summer (September–January), at 3‐day intervals. This particle release period was chosen to represent the fruiting season of the seagrass based on field observations (A. Z. Perez, *personal communication*). A fourth order Runge‐Kutta sub‐time‐stepping scheme was used to update the particle locations every hour (Feng et al., 2010). Using the random walk effect of 1 m^2^/s, particles were tracked for 7 days based on the potential dispersal duration of the seagrass fruits (Lacap et al., 2002). The grid size for tracking the particles from each sampling site was set to 500 m × 500 m. Connectivity among sampling sites was estimated as the average number of particles released from site *i* that were tracked to be in site *j* over the 7‐day dispersal duration, this ranged from 0 to 7.49 per release period, based on 48 simulation replicates in each year of the 4‐year time period. The connectivity between site *i* to site *j* was directional so the number of particles moving from site *i* to site *j* could be different to the number from site *j* to site *i* based on the currents. This directional oceanographic connectivity matrix (11 × 11 matrix) was visualized using the package *qgraph* (Epskamp et al., 2012) (Figure 1).

##### Disentangling the drivers of genetic differentiation

Spatial autocorrelation among all individuals was assessed in GenALEx using individual genetic distances and individual spatial distances based on the allelic frequencies (Smouse & Peakall, 1999). The test of spatial autocorrelation was based on 9999 random permutations and the confidence around this was determined from bootstrapping. Spatial distance categories (km) were set with endpoints of 0.01, 0.025, 0.05, 5, 10, 15, 20, 25, 30, 35, 45, and 60. Then isolation by overwater distance, oceanographic connectivity, and environmental distance were each assessed separately with the pairwise linearized *F*_ST_ matrix using a paired Mantel test of the package *vegan* in R (Oksanen et al., 2015). The pairwise matrix of overwater distance was calculated using the *marmap* package in R (Pante & Simon‐Bouhet, 2013). The pairwise (triangle) matrix of oceanographic connectivity was the total number of particles recorded at each paired site. The pairwise (triangle) matrix of environmental distance was created by converting the environmental data into a dissimilarity matrix with Gower metric using the package *cluster* 2.1.0 in R (Maechler et al., 2019).

We used variation partitioning based on partial redundancy analysis (partial‐RDA) to determine the relative contribution of geographic distance (GD), oceanographic connectivity (OC), and environmental factors (habitat characteristics, EN) in driving genetic differentiation (GS). As this analysis required both the response and the explanatory variables to be single or multicolumn numeric matrices, we transformed the “raw” data of GS, GD, OC, and EN into new data frames suitable for the analysis. First, we constructed a new matrix for the response variable (GS) by retaining all positive axes derived from a principal coordinate analysis (PCoA) on the linearized *F*_ST_ (Rousset, 1997). Second, we used spatial eigenvectors of a principal coordinates of neighborhood matrces (PCNM) on the pairwise geographic distances to construct the GD matrix. As the first three (out of eight PCNM variables) did not display collinearity with GS, those eigenvectors were used to construct the GD matrix.

For the oceanographic connectivity data frame, the pairwise matrix of oceanographic connectivity was transformed into a weighted, directed network based on graph theory using the *igraph* package in R (Csardi & Nepusz, 2015). Here, we calculated four network parameters: (i) *strength*—defined as the total amount of the weighted connection coming into and out from a sampling site (higher *strength* indicates higher degree of connectivity), (ii) *closeness*—defined as the number of steps required to access every other site from a given site, (iii) *betweenness*—the number of shortest connections between two sites that go through the site of interest, and (iv) *transitivity*—defined as the extent to which the adjacent sites of a site are connected to each other. For calculating *closeness* and *betweenness*, the package treats the connection weights as “cost” instead of “connection strength”, thus it represents the cost needed to connect nodes (higher *closeness* and *betweenness* imply a higher degree of isolation) (Barrat et al., 2004; Csardi & Nepusz, 2015). The network parameters indicated that Bathurst Island and Longitude Island were oceanographically isolated from the other sites (Table S2). The network parameters were used for the seascape genetic analysis. We ran a principal component analysis (PCA) on the centred and scaled values of the network parameters. We constructed the OC data frame based on the first three PCA axes representing 90% of the variance of the data.

For the EN data frame, we transformed the categorical variables (sediment type, habitat type, and the presence of corals) into dummy variables, and combined them with the numerical data (water depth and number of other seagrass species). Then, we ran a correspondence analysis (CA, unconstrained ordination) on the transformed environmental data. The variable most responsible in driving the environmental differentiation was sediment type. We constructed the EN data frame based on the first three CA axes from the correspondence analysis representing 97.6% of the variance of the data.

Finally, the basic formula performed in the partial RDAs was “GS ~ GD + OC + EN”. This analysis decomposed the variation in the response variable GS into components accounted for by the explanatory variable GD, OC, and EN. We calculated the adjusted *R* ^2^ to determine the amount of variation attributed to each explanatory variable controlling the effect of the other variables (the conditional effect) and without controlling the effect of the other ones (the marginal effect), and the shared fraction of variation by any combination of explanatory variables (Peres‐Neto et al., 2006). This approach is more robust to decompose spatially structured genetic variation than a Mantel test and its derived forms (Guillot & Rousset, 2013; Legendre & Fortin, 2010; Meirmans, 2015). We used the package *vegan* in R to perform the variation partitioning analysis (Oksanen et al., 2015).

## 3 RESULTS

### 3.1 Microsatellite data properties

Over the entire study area, a total of 65 alleles were observed across 16 microsatellite loci from 319 MLGs and a total of 557 samples. The marker system was able to detect MLGs from all sites with the exception of Noyon (Table 1), so this was removed from further analysis. Of the MLGs that were detected with multiple samples assigned (2–29 per MLG), the majority (70%) were not generated by chance through a unique sexual reproductive event (*P*_sex_ < 0.05) (Table 1). However, in some populations for some of the MLGs with multiple samples assigned, *P*_sex_ > 0.05 indicating that the same MLG could have arisen from a unique event. In these cases, two or three samples were generally assigned to the same MLG (Table 1). Only unique MLGs were included in further analyses. ml‐nullfreq did not detect scoring errors in all loci (estimate of genotyping error *β* < 0.001). A significant heterozygote deficit was detected in some sampling sites (Table 1). Furthermore, heterozygote deficits were detected in six loci (Thh34, Thh15, TH73, TH43, Thh1, and Thh3). ml‐nullfreq indicated the presence of null alleles in those loci, although the average frequency was <15%, which is considered relatively low (Thh34 = 0.145, Thh15 = 0.097, TH73 = 0.115, TH43 = 0.133, Thh1 = 0.116, and Thh3 = 0.120) (Chapuis & Estoup, 2007; Meeûs, 2018). After these loci were removed, the populations still showed heterozygote deficits, thus the heterozygote deficit is likely attributed to biological factors, such as inbreeding and the Wahlund effect (reduction in *H*_O_ due to subpopulation structures), rather than technical issues like the presence of null alleles (Dharmarajan et al., 2013). We retained the loci for further analysis. The test for linkage disequilibrium across multiple loci showed a small standardized index of association (*r*_D_ = 0.0217, *p* = .001), indicating a low chance of association between loci (Agapow & Burt, 2001).

Genotypic diversity (*R*) varied greatly from 0.36 to 0.94 (Table 1), although at some sites this may be an underestimate due to the possibility that MLGs may have arisen from a sexual reproductive event. The total number of observed alleles (*nA*) ranged from 24 (Bathurst Island, Bedford Island North) to 36 (Mermaid Island), while *A*_R_ ranged from 1.47 (Bedford Island—North) to 1.84 (Mermaid Island and Talon Island). The highest expected heterozygosity (*H*_NB_) was found at Longitude Island (0.216), with the lowest Sunday Island—South (0.131).

### 3.2 Genetic differentiation and connectivity

**FIGURE 2:**
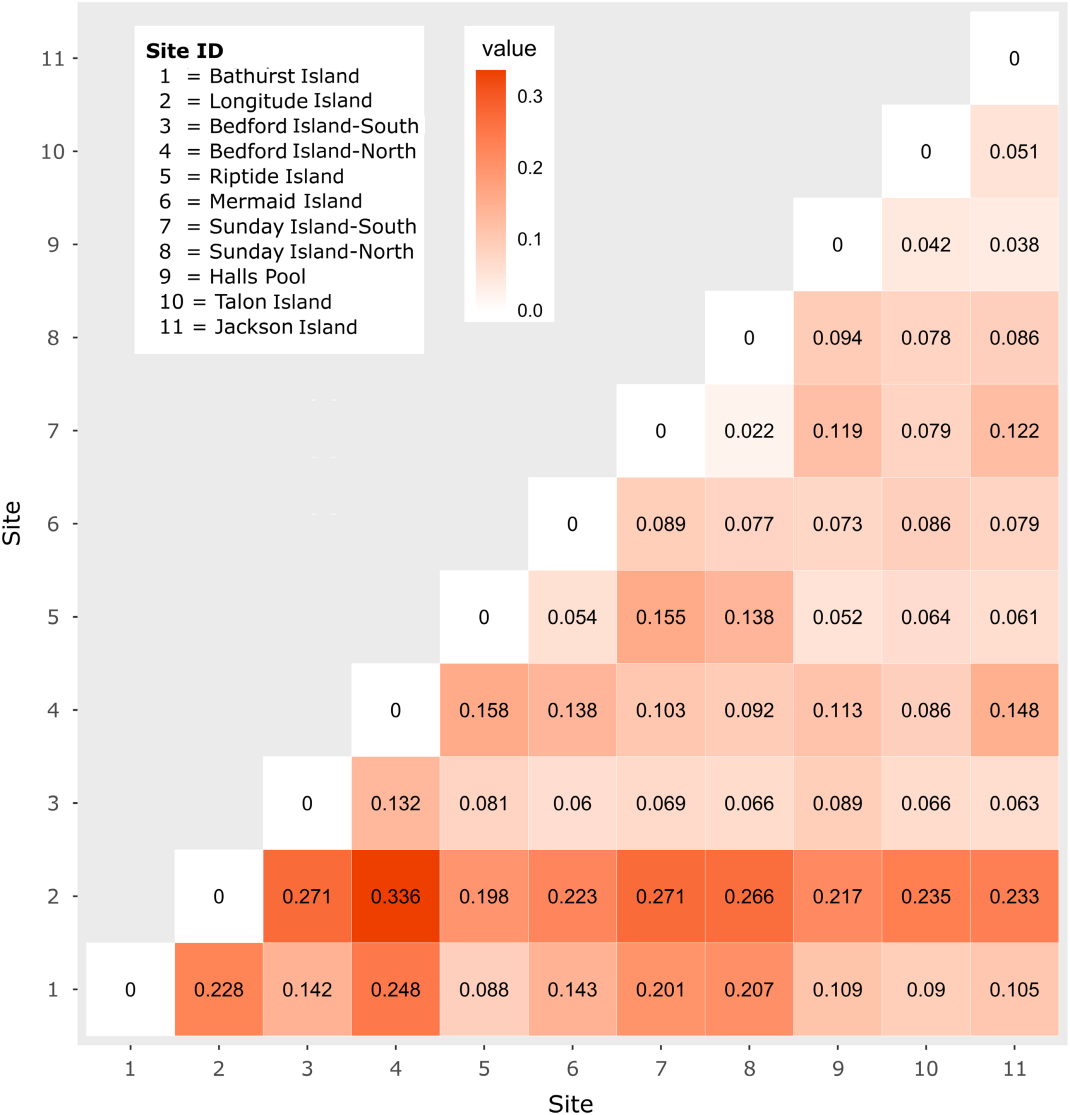
Pairwise *F*_ST_ between populations of *Thalassia hemprichii* in the Kimberley. All pairwise *F*_ST_ were significant (*p* < .01), except between the two Sunday Island populations (Sunday‐S vs. Sunday‐N; *F*_ST_ = 0.022, *p* = .066). Global *F*_ST_ = 0.22, *p* = .001.

Overall, we detected significant genetic differentiation among the sampling sites (global *F*_ST_ 0.201, *p* = .001). All pairwise *F*_ST_ were significantly greater than zero (*p* < .01), except between the two Sunday Island populations (*p* = .066) and varied by more than an order of magnitude, from 0.022 to 0.336 between Longitude Island and Bedford Island North, where sites were separated by only 12 km (Figure 2).

**FIGURE 3:**
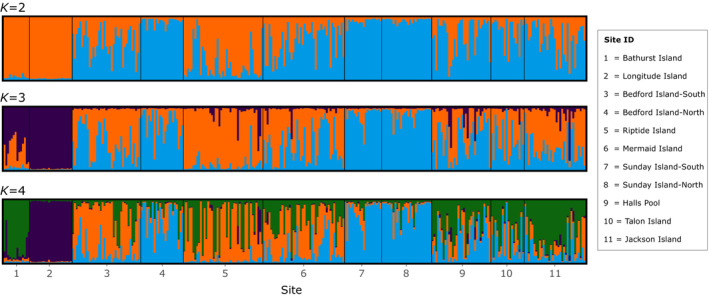
Cluster of populations resulted from structure analysis (Pritchard et al., 2000). Each individual is represented by a thin vertical line, which is partitioned into K segments that represent its estimated population group membership fractions. Each color represents a distinct population. Black lines separate individuals from geographic site locations.

**FIGURE 4:**
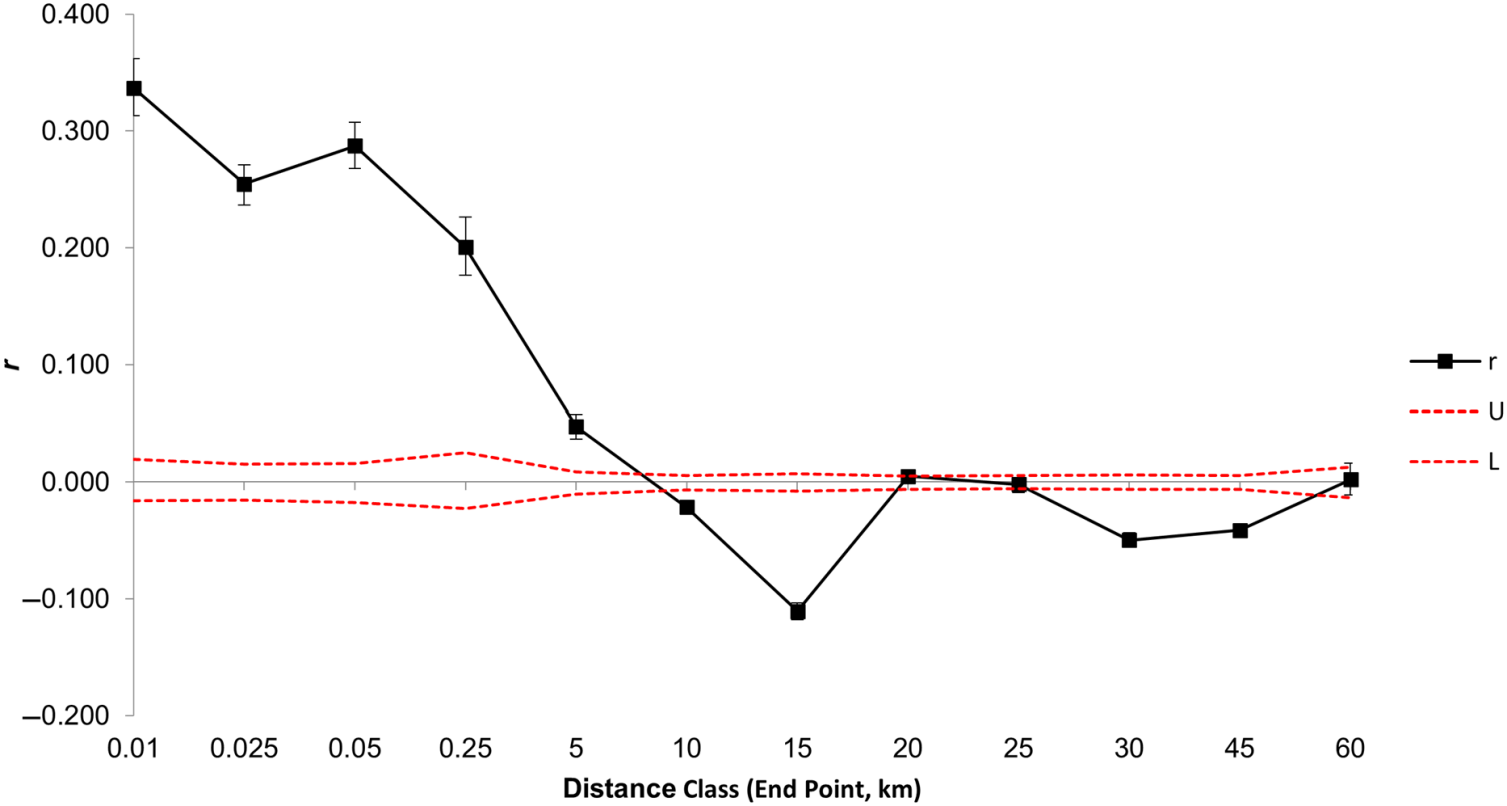
Spatial autocorrelation between individual MLGs across all sites. Samples were highly correlated over 250 m and showed significant spatial autocorrelation up to 5 km.

Bayesian probability assignment in structure revealed a spatial pattern of genetic differentiation (Figure 3). Model evaluation with the deltaK method (Evanno et al., 2005) indicated two to four populations were best supported, and *K* = 3 had the highest support. At *K* = 2, individuals from Bathurst and Longitude islands were assigned with high probability to predominantly one cluster. Individuals from the remaining sampling sites were either assigned strongly to the other cluster or exhibited a high admixture between the two clusters. At *K* = 3, individuals sampled from Bathurst and Longitude islands formed a distinct and a uniform cluster. Individuals from the remaining sites were either strongly assigned to one cluster (Sunday Island) or were highly admixed between the two remaining clusters. At *K* = 4, individuals from Bathurst Island became distinct from those at Longitude Island, but the clustering pattern of the remaining individuals did not change significantly (Figure 3). There was a strong spatial autocorrelation among individuals separated by up to 250 m and this remained significant but the strength declined when they were separated by up to 5 km (Figure 4). Beyond this distance, no significant spatial autocorrelation was detected.

### 3.3 The drivers of genetic differentiation

There was some variation in the environmental conditions among sites (Table S1). The sediments were generally coarse consisting of sand and granules, with Talon Island and Gregory Island sandier, and Bathurst Island more granular. The most northern sites, Bathurst and Longitude islands were unique in that they were not growing mixed with coral nor other seagrass species, whereas the remaining sites were. *Halophila ovalis* was at all these sites and Hall's Pool and Sunday Island North had the maximum of three other seagrass species present (*Enhalus acoroides*, *Halodule uninervis*, and *Cymodocea serrulata*). The most northern sites were also intertidal at a mean sea level of 0 m, whereas the other sites ranged from depths of 1 to 5 m below sea level (Table S1).

The highest OC was between sites in the Sunday Island group (Hall's Pool, Talon, and Jackson islands.) and the lowest OC was between these sites and the most northerly sites (Bathurst and Longitude islands) (Figure 1). The OC between the Buccaneer Archipelago sites and the intermediate sites was greater than between Sunday Island and these two regions. Within the Buccaneer Archipelago, Bathurst Island had lower OC compared to the other sites (Figure 1). Based on a paired Mantel test, there was no significant association between population level‐linearized *F*_ST_ and overwater distance (*R* = .278, *p* = .105) nor oceanographic connectivity (*R* = −.248, *p* = .938) but it was closest with environmental distance (*R* = .393, *p* = .062).

The variance partitioning analysis revealed that oceanographic connectivity and the environment were both significant drivers of genetic differentiation (Table 2). The marginal effects for oceanographic connectivity (64%) and environmental factors (62%) were significant, but geographic distance which accounted for a smaller proportion of the variation (6%) was not significant. When each individual effect was conditionally estimated by controlling the explanatory variables, the effects were not significant (*p* > .05), indicating that oceanographic connectivity and the environment factors do not explain the genetic differentiation independently but in combination.

**TABLE 2:** Variation partitioning on genetic differentiation (*F*_ST_) into components accounted for the explanatory variables: geographic distance (GD), oceanographic connectivity (OC), and environmental factors (EN).

	*R* ^2^_adj_ (%)	df_mod_	df_res_	*F*	*p‐*value
**Marginal**					
EN	61.59	3	7	6.345	.019
OC	64.39	3	7	7.028	.008
GD	5.93	3	7	1.210	.389
Residual	20.17				
**Conditional**					
EN|(OC + GD)	24.79	3	1	2.639	.299
OC|(EN + GD)	11.16	3	1	1.738	.472
GD|(OC + GD)	2.56	3	1	1.169	.534

*Note*: Fraction of variation is expressed as a percentage from *R* ^2^_adj_ values.

Abbreviations: df_res_, degrees of freedom of residuals; df_mod_, degrees of freedom of model.

## 4 DISCUSSION

### 4.1 Genetic differentiation and connectivity

The spatial setting of this study, a maximum distance between sites of 73 km, is within the potential dispersal range of this species (Lacap et al., 2002), but despite this, there was strong genetic structuring of the seagrass *T. hemprichii*. However, patterns of differentiation were idiosyncratic with respect to geographic proximity, with some of the closely situated sampling sites being the most differentiated and assigned to a different population cluster. Other population genetic studies on *T. hemprichii* have been at a much larger spatial scales, 500–1000s of kilometers (Hernawan et al., 2017; Jahnke et al., 2019; Nakajima et al., 2023; Nguyen et al., 2022; Wainwright et al., 2018) with genetic structure clear at distances of hundreds of kilometers. However, in some cases the spatial scale at which genetic differentiation manifested was similar to this study, <50 km (Jahnke et al., 2019; Nakajima et al., 2023; Nguyen et al., 2022). Interestingly, this is in stark contrast to the close relative *T. testudinum* despite both species having similar dispersal mechanisms (floating propagules and no seedbank). (Bricker et al., 2011; van Dijk et al., 2009). Significant genetic differentiation at small spatial scales is not uncommon in seagrass populations such as *Zostera marina* (Muñiz‐Salazar et al., 2006; Olsen et al., 2013; Tanaka et al., 2011), *Z. japonica* (Hodoki et al., 2013), and *Cymodocea nodosa* (Alberto et al., 2006).

### 4.2 Drivers of genetic differentiation

Our findings emphasize the potential key role of seascape attributes (oceanographic settings and environmental factors) in governing patterns of gene flow. Population differentiation is the consequence of barriers of gene flow imposed by the interactions between species’ biological traits and their environment. This is supported by a combined oceanographic connectivity and environment relationship with the patterns of gene flow revealed in our study. These mechanisms have been proposed in other locations for *T. hemprichii* (Jahnke et al., 2019; Nakajima et al., 2023; Nguyen et al., 2022) but this is the first time that they have been explicitly tested in this species. Our finding that the marginal effect of oceanographic connectivity and environmental factors were significant, but the conditional ones were not (Table 2), indicates that genetic differentiation in a complex seascape subjected to extreme tidal currents is driven by a combination of water currents constraining or facilitating dispersal combined with differential selection from the environment. In our study, the oceanographic isolation of Bathurst Island and Longitude Island is in concordance with the genetic isolation of these two sites. Furthermore, the south‐westward direction of gene flow (from the Buccaneer Archipelago to the Sunday Island group) was also in concordance with the tide‐driven current generally flowing southward into King Sound when the tide is rising, and the direction reverses toward the open ocean during the falling tide. These concordances imply that oceanographic settings can have profound consequences on gene flow in seagrass, as has been documented in other studies (e.g., Jahnke et al., 2018; Ruiz‐Montoya et al., 2015). Populations may be geographically close to each other but could still be genetically isolated if the oceanographic setting prevents dispersal (e.g., Coolen et al., 2020; Jahnke et al., 2019); or geographically distant populations can be highly connected if currents facilitate dispersal among them (e.g., Ellis et al., 2023).

Even if dispersal barriers are absent, pre‐ and/or post‐settlement selection in the recipient populations could still prevent gene flow. The significant effect of environmental characteristics observed in our study suggests the hypothesis of selection against migrants from non‐matching natal environments. The seagrass propagules may reach new locations by floating with the water current for up to 7 days, but, following arrival in the new location, the local environments may filter these migrants either through inhibiting the migrants' settlement and survival or their ability to sexually reproduce (Nosil et al., 2005; Wang & Bradburd, 2014). In our study, a significant proportion of genetic differentiation was associated with differences in environmental characteristics, especially sediment type. Sediment conditions and grain size can influence seagrass growth, survival, and species composition, possibly by controlling nutrient availability and providing a physical matrix for the roots and rhizomes to anchor (Short, 1987; Tanaka & Kayanne, 2007; Terrados et al., 1999; van Katwijk & Wijgergangs, 2004). This scenario has also been proposed for this species in Vietnam where there was strong genetic differentiation between sites with hard versus soft substrates and the intertidal mussel *Perna perna* (Zardi et al., 2011). To determine whether local adaptation/selection truly drives genetic differentiation, one could examine population‐specific fitness across different sediment types in reciprocal transplants, multiple common garden, or provenance trial experiments (Wang & Bradburd, 2014). In addition, genetic studies employing a larger panel of markers at functional loci would provide better statistical resolution to examine the hypothesis of local adaptation/selection (Tiffin & Ross‐Ibarra, 2014).

Beside the local adaptation/selection scenario, it is also possible that a reduced effective gene flow among populations observed in our study was partially due to colonization history (founder events). Significant heterozygote deficits observed in some sampling sites might be a microevolutionary consequence of founder events as the founder population generally represent a small proportion of the genetic variation from a larger source population (Mayr, 1963). Under this scenario, the first few founders to colonize available habitats bring small, but sufficient genetic variation. These founders monopolize the habitat and prevent settlement of new migrants. Consequently, the gene frequency is resistant to decay of genetic exchange, leading to genetic differentiation (De Meester et al., 2002; Orsini et al., 2013).

Our study revealed that geographic distance poorly explained the pattern of gene flow. At a small spatial scale, within the dispersal range of the organism and in a system with multidirectional currents, a stepping‐stone model of dispersal, in which gene flow is limited only by geographic distance, is not likely to occur, as observed in the coral, *Acropora spicifera*, Houtman Abrolhos Islands (Thomas et al., 2015) and the whelk, *Kelletia kelletii*, Santa Barbara Channel (White et al., 2010). In contrast, the significant effect of geographic distance in limiting gene flow is often found at a larger spatial scale in marine systems, for example, in a series of local populations current along the coast (Couceiro et al., 2007; Ellis et al., 2023; Thiel & Haye, 2006).

From an analytical standpoint, we highlight the integration of physical and environmental data into population genetic studies to fully understand processes governing gene flow. Oceanographic connectivity data generated from biophysical simulation is potentially very useful to predict population connectivity in the absence of genetic data. Our seascape approach can be applied elsewhere where genetic studies are lacking. For terrestrial settings, a similar approach using a biophysical simulation based on wind‐mediated dispersal of pollen and seed has also improved the understanding of processes influencing gene flow (Kling & Ackerly, 2021; Kuparinen et al., 2007; Schueler & Schlunzen, 2006).

## 5 CONCLUSIONS

This study presents evidence of significant spatial genetic differentiation among populations of the seagrass, *T. hemprichii*, over a relatively small spatial scale that could not be explained by geographic distance. A seascape genetic approach showed that oceanographic connectivity, in combination with environmental factors, explained the patterns in genetic differentiation, and the effects of these components cannot be separated. Our findings add to the growing evidence for the significant contribution of oceanography and environmental factors in governing the pattern of genetic differentiation in marine populations and that survival of marine species is a complex interaction between connectivity among populations of a species and environment. Our expectations of high genetic connectivity in a species with floating propagules and a capacity for LDD were not met. Instead, we encountered high levels of genetic differentiation among populations tens of kilometers apart and populations are possibly locally adapted, suggesting a local conservation management program to be more appropriate. As the environment rapidly changes under anthropogenic‐driven climate change, populations are at risk, even those with high levels of connectivity (Bernhardt & Leslie, 2013). Clearly, understanding population connectivity should be a priority for marine conservation and management.

## AUTHOR CONTRIBUTIONS

**Udhi Hernawan:** Conceptualization (equal); data curation (lead); formal analysis (lead); funding acquisition (supporting); investigation (equal); methodology (equal); project administration (supporting); resources (equal); software (equal); supervision (supporting); validation (equal); visualization (equal); writing – original draft (equal); writing – review and editing (equal). **Kor Jent van Dijk:** Investigation (supporting); methodology (supporting); resources (supporting); software (supporting); supervision (supporting); writing – review and editing (supporting). **Gary Andrew Kendrick:** Conceptualization (supporting); investigation (supporting); supervision (supporting); writing – review and editing (supporting). **Ming Feng:** Formal analysis (supporting); methodology (supporting); resources (supporting); validation (supporting); writing – review and editing (supporting). **Oliver Berry:** Conceptualization (supporting); formal analysis (supporting); investigation (supporting); methodology (supporting); resources (supporting); writing – review and editing (supporting). **Christopher Kavazos:** Formal analysis (supporting); writing – review and editing (supporting). **Kathryn McMahon:** Conceptualization (lead); data curation (equal); formal analysis (equal); funding acquisition (lead); investigation (equal); methodology (equal); project administration (lead); resources (lead); software (equal); supervision (lead); validation (equal); visualization (equal); writing – original draft (equal); writing – review and editing (equal).

## CONFLICT OF INTEREST STATEMENT

None declared.

## Supporting information


Figure S1

Appendix S1

## Data Availability

Microsatellite data and GPS coordinates are available from ECU's online data repository (https://ro.ecu.edu.au/datasets/31/) and R script is available at BRIN's scientific data repository (https://data.brin.go.id/dataverse/Th‐Kimberley).
