# Supplementary material for: Ocean connectivity and habitat characteristics predict population genetic structure of seagrass in an extreme tropical setting
Source: Ecol Evol. 2023 Jul 3;13(7):e10257. doi: 10.1002/ece3.10257 (PMC10316484; doi:10.1002/ece3.10257)
Supplement: Supplementary file 2 — Appendix S1 [file ECE3-13-e10257-s002.docx]

**Appendix Table S1**. Environmental data used to calculate environmental distance and for incorporation into the mantel test and the partial-RDA analysis. Water depth was measured relative to mean sea level (m). Long: longitude; Lat: Latitude; Ho: *Halophila ovalis*; Hu: *Halodule uninervis*; Ea: *Enhalus acoroides*; Cs: *Cymodocea serrulata*.

| **Island Group** | **Site name** | **Long.** | **Lat.** | **Water depth** | **Sediment type** | **Habitat type** | **Coral** | **No. other seagrass species present** | **Ho** | **Hu** | **Ea** | **Cs** |
| --- | --- | --- | --- | --- | --- | --- | --- | --- | --- | --- | --- | --- |
| Buccaneer Archipelago | Bathurst Is. | 123.52317 | -16.04164 | 0 | Granule | Reef_terrace | No | 0 | No | No | No | No |
|  | Longitude Is. | 123.39378 | -16.06936 | 0 | Sand-granule | Reef_lagoon | No | 0 | No | No | No | No |
|  | Bedford Is. South | 123.34789 | -16.16476 | 4 | Sand-granule | Reef_terrace | Yes | 2 | Yes | Yes | No | No |
|  | Bedford Is. North | 123.29884 | -16.13672 | 1 | Sand-granule | Reef_terrace | Yes | 2 | Yes | No | Yes | No |
| North eastern King Sound | Riptide Is. | 123.302816 | -16.303502 | 4 | Sand | Reef_lagoon | Yes | 1 | Yes | No | No | No |
|  | Mermaid Is. | 123.342706 | -16.432222 | 5 | Sand-granule | Reef_lagoon | Yes | 2 | Yes | Yes | No | No |
| Sunday Is. Group | Sunday_Is. South | 123.19805 | -16.42537 | 1 | Sand-granule | Reef_terrace | Yes | 2 | Yes | No | Yes | No |
|  | Sunday_Is. North | 123.21033 | -16.39642 | 3 | Sand-granule | Reef_terrace | Yes | 4 | Yes | Yes | Yes | Yes |
|  | Halls Pool | 123.16699 | -16.41813 | 1 | Sand-granule | Reef_terrace | Yes | 4 | Yes | Yes | Yes | Yes |
|  | Talon Is. | 123.135 | -16.40183 | 3 | Sand | Reef_lagoon | Yes | 2 | Yes | No | Yes | No |
|  | Jackson Is. | 123.10225 | -16.44053 | 1 | Sand-granule | Reef_terrace | Yes | 2 | Yes | No | Yes | No |
|  | Noyon | 123.06940 | -16.43792 | 1 | Sand | Reef lagoon | Yes | 1 | Yes | No | No | No |
|  | Shenton Bluff | 123.04702 | -16.48246 | 2 | Sand-granule | Reef lagoon | Yes | 1 | Yes | No | No | No |

**Appendix Table S2.** Network parameters constructed using the package *igraph* from the oceanographic connectivity between the sampling sites. Definition of the network parameters can be found in the Materials and Methods. Note that, the package treats the connection weights as ‘cost’ instead of ‘connection strength’ for calculating *closeness* and *betweenness*, thus the parameter values represent the cost needed to connect nodes.

| **Site ID** | **Site** | **Strength** | **Closeness** | **Transitivity** | **Betweenness** |
| --- | --- | --- | --- | --- | --- |
|  |  |  |  |  |  |
| 1 | Bathurst Is. | 4.70 | 0.74 | 1.31 | 50 |
| 2 | Longitude Is. | 10.26 | 0.71 | 0.70 | 33 |
| 3 | Bedford Is. –South | 15.96 | 0.78 | 0.95 | 1 |
| 4 | Bedford Is. – North | 19.68 | 0.35 | 1.12 | 0 |
| 5 | Riptide Is. | 20.19 | 0.70 | 1.06 | 15 |
| 6 | Mermaid Is. | 16.53 | 0.66 | 0.96 | 0 |
| 7 | Sunday Is. –South | 20.32 | 0.37 | 1.03 | 1 |
| 8 | Sunday Is. –North | 18.46 | 0.34 | 0.83 | 11 |
| 9 | Halls Pool | 27.29 | 0.59 | 0.93 | 21 |
| 10 | Talon Is. | 25.31 | 0.61 | 0.87 | 12 |
| 11 | Jackson Is. | 22.04 | 0.42 | 0.80 | 4 |
| 12 | Noyon | 17.13 | 0.36 | 1.07 | 2 |
| 13 | Shenton Bluff^++^ | 18.81 | 0.52 | 0.76 | 0 |

**Figure S1.** The relationship between *G*_ST_ and *H*_S_ estimated from 16 microsatellites in 11 populations of *Thalassia hemprichii* in the Kimberley. Each triangle shows the pair of *G*_ST_ and *H*_S_ values estimated from a single marker, and the blue line shows the fitted regression of *G*_ST_ and *H*_S_ (*R*^2^=0.07458, p-value= 0.3061). The correlation coefficient between *G*_ST_ and *H*_S_ is 0.2732 (p-value =0.3059).
